# Intrathecal morphine versus transversus abdominis plane block for caesarean delivery: a systematic review and meta-analysis

**DOI:** 10.1186/s12871-021-01392-9

**Published:** 2021-06-22

**Authors:** Tao-ran Yang, Xue-mei He, Xue-han Li, Ru-rong Wang

**Affiliations:** Department of Anesthesiology, The Research Units of West China (2018RU12), Chinese Academy of Medical Sciences, West China Hospital, Sichuan University, No. 37, Guoxue Xiang, Chengdu, 610041 Sichuan China

**Keywords:** Intrathecal morphine, Transversus abdominis plane block, Caesarean delivery

## Abstract

**Background:**

The number of caesarean deliveries has been increasing. Although intrathecal morphine (ITM) can relieve pain and is widely applied in caesarean deliveries, it is associated with many side effects. Transversus abdominis plane block (TAPB), a new analgesic technology, has also began playing a certain role after caesarean delivery, with fewer adverse effects. This study mainly compares the analgesic and adverse effects of ITM and TAPB in caesarean delivery.

**Methods:**

We systematically searched PubMed, Cochrane Library, EMBASE, and Web of Science, for randomised controlled trials (RCTs) published before 9 October, 2020 to compare the effects of ITM and TAPB. Primary outcome of the study was the pain score at rest 24 h after caesarean delivery, whereas the secondary outcomes were the pain score at movement 24 h after operation, postoperative nausea and vomiting (PONV), itching, and morphine consumption. For the outcome assessment, we conducted a sensitivity analysis.

**Result:**

Six RCTs involving 563 patients and meeting the study inclusion criteria were included in this study. Results indicated no significant difference in the pain score between ITM and TAPB at 24 h of rest or movement. The sensitivity analysis results indicated that the resting pain score (95% CI = − 1.27 to − 0.28; *P* = 0.002) and 24-h moving pain score (95% CI = − 1.8 to − 0.07; *P* = 0.03) of the ITM group were lower than those of the TAPB group. The consumption of morphine in the ITM group was lower than in the TAPB group (95% CI = 1.92 to 4.87; *P* < 0.00001); however, in terms of adverse reactions, the incidence of pruritus (95% CI = 1.17 to 8.26; *P* = 0.02) and PONV (95% CI = 1.92 to 4.87, *P* < 0.00001) in the ITM group was higher than in the TAPB group.

**Conclusion:**

Parturients in the ITM and TAPB groups exhibited similar analgesic effects. However, in the sensitivity analysis performed by eliminating the studies causing heterogeneity, the ITM group was found to have superior analgesic effects compared with the TAPB group, with less morphine consumption. Differently, the TAPB group displayed less side effects such as PONV. Therefore, TAPB is still a valuable analgesia option for patients who cannot use ITM for analgesia after caesarean delivery or those having a high risk of PONV.

**Trial registration:**

Registration number: Registered on Prospero with the registration number of CRD42020210135.

**Supplementary Information:**

The online version contains supplementary material available at 10.1186/s12871-021-01392-9.

## Introduction

The rate of caesarean delivery has been increasing annually because of social and psychological reasons [1]. Postoperative pain not only brings psychological torture to patients but also has a certain degree of impact on the recovery of patients after surgery and wound recovery [2, 3]. For parturients after caesarean delivery, the lack of analgesia affects the maternal postpartum recovery, breast-feeding, and baby development on the hand, whereas on the other hand, it increases the risk of postpartum depression [4, 5]. Approximately 500,000 women in Europe have been reported to experience acute postoperative pain annually [3]. Therefore, exploring effective analgesic methods for parturients after caesarean delivery is essential.

Intrathecal morphine (ITM) is considered the “gold standard” for providing analgesia after caesarean delivery. ITM can make hydrophilic morphine easily reach the cerebrospinal fluid and rapidly act on the central nervous system [6]. Therefore, the use of ITM can provide a superior analgesic effect after caesarean delivery compared with the systemic opioid analgesia technique [7, 8]. Although ITM has obvious analgesic advantages, its side effects such as nausea, vomiting, itching, and even respiratory depression restrict its further application [9, 10].

With the rapid development of the ultrasound technology, the use of transversus abdominis plane block (TAPB) in regional anaesthesia is becoming increasingly popular. Local anesthetics are mainly injected between the superficial layer of transversus abdominis plane and deep layer of internal oblique muscle, thus blocking the anterior abdominal wall afferent nerve of T6-L1 [11]. Recent studies have indicated that TAPB may play a vital role as an effective pain block of somatic surface pain induced by incision, which is much more obvious than the visceral pain caused by the traditional transverse incision [12, 13]. According to the newest PROSPECT guideline, TAPB improve pain relief, increase patient satisfaction, and result in a reduction of rescue analgesia; the potential side effects of these regional analgesic techniques are also limited, and therefore, their use is recommended for providing analgesia to patients [14].

Some meta-analyses on patient-controlled intravenous analgesia, quadratus lumborum block, and intrathecal morphine injection are available in literature. However, most of these studies have discussed the analgesic effect of a combination of TAPB and ITM. Some researchers believe that in case of postoperative analgesia with ITM, the addition of TAPB cannot further alleviate the pain. Only one meta-analysis compared the analgesic effect of TAPB and ITM after caesarean delivery [15]. Results of this meta-analysis indicated that ITM produces a superior analgesic effect compared with TAPB at rest 24 h after surgery; however, the evidence was not convincing since only two RCTs were included in the meta-analysis. In the present study, we aimed to synthesize the available data through updated systematic review and meta-analysis to assess, whether the analgesic effect and side effects of ITM differ from those of TAPB after caesarean delivery.

## Methods

This systematic review and meta-analysis was conducted in accordance with the newest PRISMA recommendation [16] and was registered on Prospero (number: CRD420210135). Two researchers (YTR and HXM) searched PubMed, Cochrane Library and EMBASE from inception to 9 October, 2020 without restriction on language and region of publication. The PRISMA checklist is provided in Additional file 1.

A comprehensive search strategy by using relevant search terms, which were selected from Medical Subject Headings, EMBASE Subject Headings, and Entry terms, was employed. The databases were explored using a search algorithm with Boolean operators: ‘(transversus abdominis plane block OR transversus abdominis block OR abdominal muscle block OR TAP) AND (spinal Injections OR intrathecal injections OR intraspinal injection OR ITM) AND (caesarean section OR caesarean delivery OR abdominal deliveries OR C Section OR postcaesarean section)’.

### Study selection

The determination of inclusion and exclusion criteria for selection of studies preceded our meta-analysis. Inclusion criteria were as follows: study participants comprising adult female; patients with American Society of Anesthesiologists grade ≤ 3; spinal anesthesia being the preferred mode; parturients with caesarean section/delivery; and pfannenstiel incision being the surgical approach. Exclusion criteria were as follows: observational or retrospective study; patients’ BMI ≥ 40; and study participants having a history of drug allergy and opioid tolerance. Two researchers (YTR and HXM) selected the studies meeting the inclusion criteria for full-text reading by reviewing the title and abstract. Differences at any time point were resolved by a third researcher (WRR). The authors then performed additional literature searches of the clinical trials registry (www.clinicaltrials.gov).

### Data extraction

The two researchers (YTR and HXM) independently extracted the following data (Table 1): number of participants; age; weight; drugs; analgesic methods of the control and the intervention group; methods of anaesthesia; and additional medications. Discrepancies were resolved through consensus or, if necessary, through discussion with the third author (WRR).

**Table 1:** Characteristics of included studies

Author, year	Total Sample	Methods	Age	Weight	Sample	Drug	Anaesthesia	Additional drug
Kwikiriza,2019	*n* = 130	ITM	24.7 (5.6)	65.2 (11.9)	*n* = 65	Morphine 0.1 mg	Spinal anaesthesia with bupivacaine 10 mg	Diclofenac 50 mg and paracetamol 1 g every 8 h
		TAPB	24.8 (4.8)	63.9 (12.5)	n = 65	Bupivacaine 75mg		
Kanazi,2010	*n* = 57	ITM	33 (6)	82 (13)	*n* = 28	Morphine 0.2 mg	Spinal anaesthesia with bupivacaine 12.75 mg	Rectal diclofenac 100 mg every 12 h and intravenous acetaminophen 1 g every 6 h
		TAPB	30 (5)	78 (16)	*n* = 29	Bupivacaine 75mg		
McMorrow,2011	*n* = 40	ITM	33 (4)	70 (13)	*n* = 20	Morphine 0.1 mg	Spinal anaesthesia with bupivacaine 11–12.5 mg and 10μg fentanyl	Rectal diclofenac 100 mg every 18 h and oral acetaminophen 1 g every 6 h
		TAPB	33 (5)	72 (14)	n = 20	Bupivacaine 75mg		
Dereu,2019	*n* = 181	ITM	34 [31 to 38]	74 [67 to 83]	*n* = 89	Morphine 0.1 mg	Spinal anaesthesia with bupivacaine 10 mg and fentanyl 25μg and epinephrine 100μg	Paracetamol orally 1 g every 6 h and ibuprofen 600 mg every 8 h
		TAPB	34 [30.75 to 37]	74.5 [67 to 82.25]	*n* = 92	Bupivacaine 150 mg and clonidine 75μg		
Loane,2012	*n* = 66	ITM	35 (3)	81 (13)	*n* = 33	Morphine 0.1 mg	Spinal anaesthesia with bupivacaine 11.25 mg and fentanyl 10 μg	Naproxen orally or rectal 500 mg every 12 h and acetaminophen 1 g every 6 h
		TAPB	34 (5)	78 (12)	n = 33	Ropivacaine 3 mg/kg, no more than 200 mg		
Jarraya,2016	*n* = 86	ITM	33.24 (5.7)	75.63 (11.7)	*n* = 43	Morphine 0.1 mg	Spinal anaesthesia with bupivacaine 10 mg and fentanyl 2.5μg	Paracetamol orally 1 g of every 6 h and ketoprofen 100 mg and tramadol 100 mg every 12 h
		TAPB	32.83 (6.1)	76.63 (10.4)	n = 43	Ropivacaine 60 mg		

The number represents the mean (standard deviation) or median (interquartile range) *ITM* intrathecal morphine; *TAPB* transversus abdominis plane block

Data were extracted for synthesis either directly from the paper through extrapolation from graphs by using Plot digitizer (http://www.plotdigitizer.sourceforge.net) or by contacting the corresponding authors for the required data, if direct extraction was not possible. We extracted continuous results as the mean and standard deviation. If no direct data were available in the original text, we extracted the data from the graph. If the median was displayed, we used Hozo and other formulas to convert the median and range into mean and standard deviation [17].

Quality of the reviewed trials was assessed using the Cochrane Risk of Bias tool independently by two of the authors (YTR and HXM).

### Outcome

We considered the pain score at rest 24 h after operation as the primary outcome and converted the evaluation criteria of 0–100 points into 0–10 points for the analysis. If the data in the original article reported both the visceral and somatic scores, we selected the higher of the two pain scores for the data analysis. The pain score at movement 24 h after operation, PONV, incidence of itching, and morphine consumption were considered the secondary outcomes.

Postoperative morphine requirement was compared between the groups. Other forms of opiate analgesia were converted into intravenous morphine equivalents as follows: oral tramadol (1: 20), parenteral fentanyl (100: 1), and intravenous oxycodone (1: 1) [18, 19].

### Data analysis

Continuous variables are presented as the 95% confidence interval and mean difference (MD). For presenting dichotomous variables, the odds ratio (OR) is used. The I^2^ value was used to evaluate the heterogeneity between the studies, and I^2^ of values > 50% suggested significant heterogeneity between the studies [20]. For continuous data, we determined MD. For assessing the outcome, we performed a sensitivity analysis by using the leave-one-out approach to identify the possible sources of heterogeneity. All statistical analyses were performed in Review Manager 5.3.

### Risk of bias assessment

Two authors (YTR and HXM) independently assessed the methodological quality of each study by using the Cochrane Collaboration Risk of Bias tool for RCTs [21]. This tool can be used to assess random sequence generation (selection bias), allocation concealment (selection bias), blinding of participants and personnel (performance bias), blinding of outcome (detection bias), incomplete outcome data (attrition bias), selective reporting (reporting bias), and other biases. The risk of bias was classified as high, low, and unclear. Disagreements between the two reviewers regarding the overall risk of bias assessment were resolved through discussion and consensus.

## Result

**Fig. 1:**
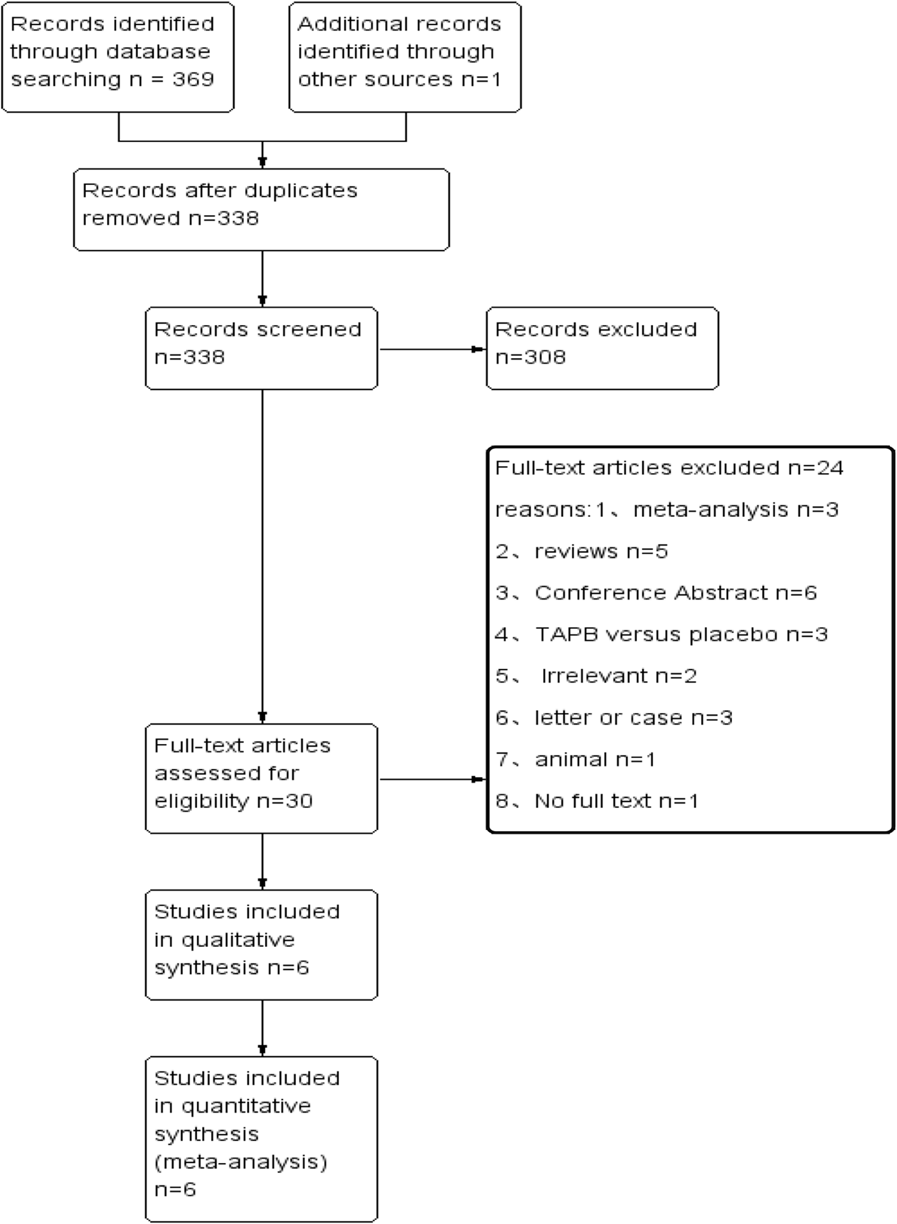
Flow chart showing selection of articles for review

A total of 369 studies were identified through the systematic database search. After screening for duplicate studies, we obtained 338 records. Thirty full-text publications were assessed for eligibility after reviewing the title and abstract. We excluded 24 studies because the participants, interventions, or outcomes did not meet our inclusion criteria or because the study was not an RCT. Finally, we included a total of 6 RCTs for the analysis [22–27]. Figure 1 depicts a flow chart of the study selection process.

### Study characteristics

Table 1 summarises the characteristics and outcomes of the 6 RCTs selected according to the inclusion criteria. All included studies were published before October 2020. The sample size of these studies ranged from 40 to 180. Most of the included RCTs had used 10 mg–12.75 mg bupivacaine with or without opioid for spinal anaesthesia, and one [25] of these studies had used epinephrine additionally in the bupivacaine for spinal anaesthesia. In all the studies, the use of 0.1 mg–0.2 mg morphine in the ITM group was documented. For local blockade in the TAPB group, four RCTs [22–25] had used bupivacaine, whereas the other two trials [26, 27] had used ropivacaine. In a study comparing ITM and TAPB [25], 75 μg clonidine added as an adjuvant in combination with ropivacaine was administered to the TAPB group.

### Quality assessment

**Fig. 2:**
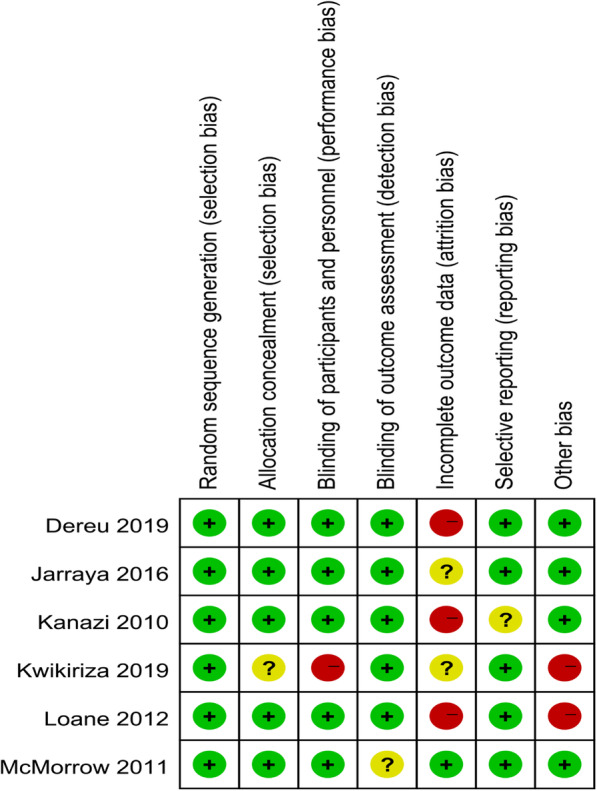
Quality assessment of included trials. Green circle = low risk of bias; red circle = high risk of bias; yellow circle = unclear risk of bias

Figure 2 presents the risk of bias of all the RCTs. According to the Cochrane Risk of Bias tool, four trials [22, 23, 25, 26] were found to have a high risk of bias. Of all the trials, 3 trials [23, 25, 26] exhibited a high risk of attrition bias, 2 trials [22, 26] exhibited a high risk of other bias, and one trial [22] exhibited a high risk of selection bias. Unclear risk of bias was documented in four trials [22–24, 27] owing to the selection bias, detection bias, attrition bias, or reporting bias.

### Primary outcome

#### Pain scores at rest 24 h after operation

**Fig. 3:**
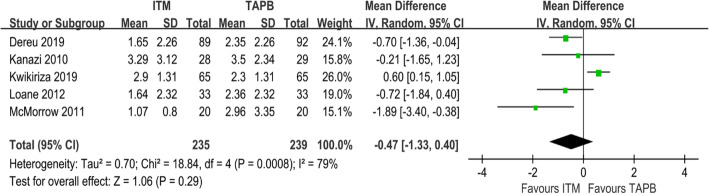
Forest plot showing pain scores at rest 24 h after surgery

Five studies involving 474 patients had reported pain scores at rest 24 h after surgery [22–26]. No significant difference was noted in the pain score between the ITM and TAPB groups (MD = − 0.47; 95% CI = − 1.33 to 0.40; I^2^ = 79%; *P* = 0.29) (Fig. 3). Results of the sensitivity analysis are presented in Table 2. The direction of the primary outcome was found to change after the RCT by Kwikiriza was excluded. The results indicated that the pain score of the ITM group was lower than that of the TAPB group, indicating that ITM produces superior analgesic effects than TAPB (MD = − 0.77; 95% CI = − 1.27 to − 0.28; I2 = 0%; *P* = 0.002), which further indicated that the meta-analysis has poor robustness. The source of bias is further analysed in the discussion section.

**Table 2:** The sensitivity analysis of pain scores at rest 24 h after surgery

Study	statistics with study removed					
	MD	Lower limit	Upper limit	Z value	***P*** value	I^**2**^ value
Dereu 2019 [25]	−0.43	−1.54	0.68	0.76	0.45	77%
Kanazi 2010 [23]	−0.54	−1.55	0.48	1.04	0.3	84%
Kwikiriza 2019 [22]	−0.77	− 1.27	− 0.28	3.05	0.002	0%
Loane 2012 [26]	−0.43	−1.45	0.6	0.81	0.42	83%
McMorrow 2011 [24]	−0.2	−1.03	0.63	0.48	0.63	76%

*MD* mean difference

### Secondary outcome

#### Pain scores at movement 24 h after surgery

**Fig. 4:**
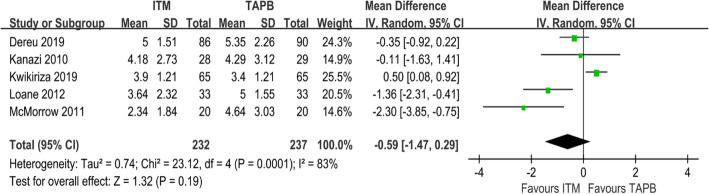
Forest plot showing pain scores at movement 24 h after surgery

Five studies involving 474 patients had reported pain scores at movement 24 h after surgery [22–26]. No significant difference was observed in the pain score between the ITM and TAPB groups (MD = − 0.59; 95% CI = − 1.47 to 0.29; I^2^ = 83%; *P* = 0.19) (Fig. 4). Results of the sensitivity analysis are summarised in Table 3. After the exclusion of Kwikiriza’s RCT, the pain score of the ITM group was found to be lower than that of the TAPB group (MD = − 0.94; 95% CI = − 1.80 to − 0.07; I^2^ = 62%; *P* = 0.03), although heterogeneity between the studies was still observed. After discussion, we concluded that a high risk of attrition bias in a study [25] may be the cause of heterogeneity.

**Table 3:** The sensitivity analysis of pain scores at movement 24 h after surgery

Study	statistics with study removed					
	MD	Lower limit	Upper limit	Z value	P value	I2 value
Dereu 2019 [25]	−0.74	−2.07	0.6	1.08	0.28	86%
Kanazi 2010 [23]	−0.69	−1.7	0.31	1.35	0.18	87%
Kwikiriza 2019 [22]	−0.94	−1.8	−0.07	2.13	0.03	62%
Loane 2012 [26]	−0.37	−1.27	0.54	0.79	0.43	80%
McMorrow 2011 [24]	−0.28	−1.09	0.53	0.68	0.5	80%

*MD* mean difference

### PONV 24 h

**Fig. 5:**
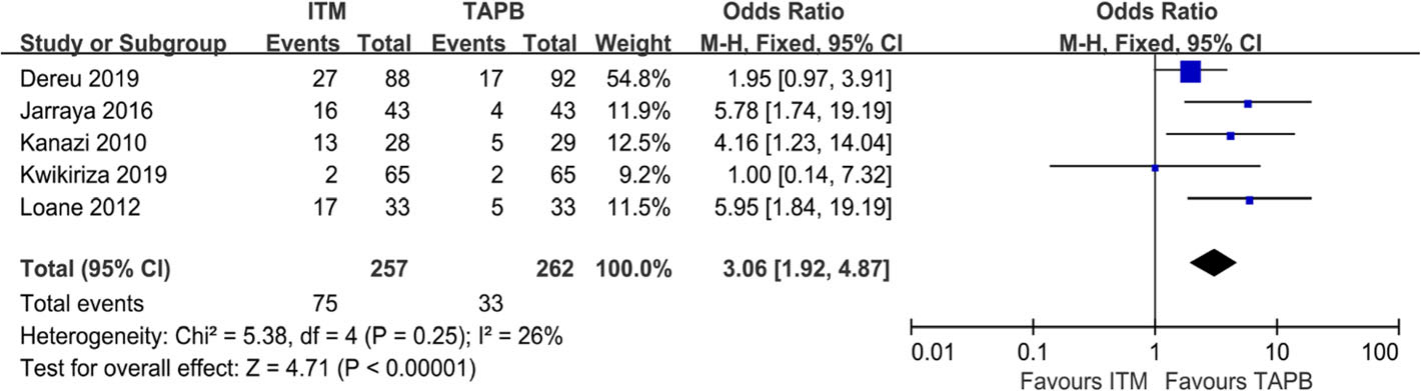
Forest plot showing the incidence of postoperative nausea and vomiting after surgery

The incidence of PONV was recorded in five articles, with a total of 519 (were reported) cases (Fig. 5) [22, 23, 25–27]. In these trials, higher incidence of postoperative nausea and/or vomiting had been reported in the ITM group than in the TAPB group, and the difference was statistically significant (OR = 3.06; 95% CI = 1.92 to 4.87; *P* < 0.00001; Fig. 5), with mild heterogeneity (I2 = 26%).

### Morphine consumption

**Fig. 6:**
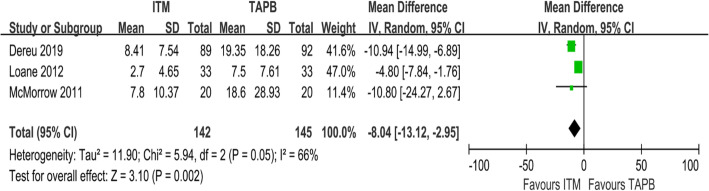
Forest plot showing the consumption of morphine 24 h after surgery

A total of 287 people had reported to have consumed morphine 24 h after operation (Fig. 6) [24–26]. Morphine consumption between the ITM and TAPB groups was found to differ significantly (MD = − 8.04; 95% CI = − 13.12 to − 2.95; I^2^ = 66%; *P* = 0.002), which indicated that ITM analgesia causes a certain reduction in the postoperative morphine demand compared with that in the TAPB group.

### Itching 24 h after surgery

**Fig. 7:**
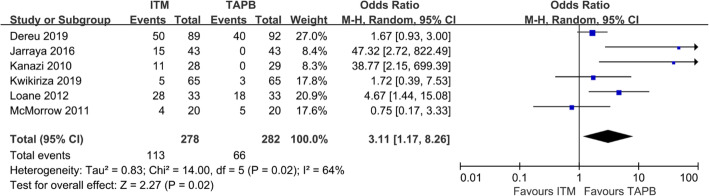
Forest plot showing the incidence of itching after surgery

Pruritus 24 h after surgery was reported in 6 studies involving 560 patients (Fig. 7) [22–27]. In one study [24], patients were asked to subjectively express whether itching or not, and the itching were graded. However, the degree of itching was not evaluated in other studies and only the number of pruritus requiring treatment was reported, which cause the high heterogeneity. Therefore, we discarded the number of people with itching that did not require treatment in this study [24], and the incidence of itching was found to be higher in the ITM group than in the TAPB group (OR = 3.11; 95% CI = 1.17 to 8.26; *P* = 0.02) (Fig. 7), that may be a source of heterogeneity.

## Discussion

In this systematic review and meta-analysis, the results of six RCTs were included to compare the analgesic effects of ITM and TAPB after caesarean delivery. The results showed no significant difference in pain scores between the ITM and TAPB groups. However, a high heterogeneity was observed across the included RCTs. Therefore, we conducted a sensitivity analysis, and after excluding a study, we found that the analgesic effect of ITM is superior to that of TAPB at rest and movement. In addition, the demand for morphine in the ITM group was found to be lesser than in the TAPB group. Although no significant difference was observed in the incidence of postoperative pruritus between the ITM and TAPB groups, the incidence of PONV was higher in the ITM group than in the TAPB group.

None of the meta-analyses have individually and systematically compared the analgesic effects of ITM and TAPB after caesarean delivery yet. Among the existing analgesic methods used after caesarean delivery, ITM is undoubtedly the first choice of anesthesiologists [28]. Although morphine can produce relatively superior analgesic effects, its adverse reactions (such as nausea, vomiting and respiratory depression) have limited its application [9]. Studies have found that morphine can be detected in the breast milk of mothers who use ITM for analgesia, which may affect newborns. Although the increased incidence of adverse events in infants during the maternal morphine treatment has not been reported, close monitoring of infants is indispensable [29]. Therefore, researchers are exploring an optimal analgesic method. With the rapid development of the ultrasound technology, ultrasound-guided TAPB has attracted extensive attention. The traditional pfannenstiel incision is chosen as the surgical approach, and its area is within the range of T6-L1 that can be blocked by TAPB, which also provides a theoretical basis for using TAPB for analgesia after caesarean section [30].

Previously, one meta-analysis was conducted to compare ITM and TAPB for multimodal analgesia after caesarean delivery [15]; however, this study focused on investigating the analgesic effects of a combination of TAPB and ITM. This meta-analysis indicated that the parturients who receive ITM and TAPB display a slight blocking effect and only the early postoperative movement pain score could be reduced. In another meta-analysis by Mishriky, although the authors had compared the analgesic effects between ITM and TAPB further analyses could not be performed and conclusions could not be derived because of the small size and excessive bias among the included studies. In the present meta-analysis, we included sufficient number of studies, evaluated the quality of included studies, compared the effects of ITM and TAPB for postcaesarean analgesia through various outcomes, and analysed the heterogeneity across studies.

In this meta-analysis, multiple outcome indicators suggest excessive heterogeneity. Using the leave-one-out method, we identified Kwikiriza’s RCT [22] as the source of heterogeneity across the studies. Possible reasons for the heterogeneity are as follows: 1. Kwikiriza’s RCT was conducted in Uganda, which is a poverty-stricken region in Africa, with limited postoperative care and resources. In this region, mothers do not receive postoperative analgesia on time, and cannot complete postoperative follow-up independently. 2. Most of the mothers in this region possess a low level of education and perceive pain as common problem; they do not actively report mild pain unless being repeatedly asked by researchers, which causes biases in the reported results.

In our meta-analysis, parturients in the ITM group displayed superior analgesic effects and less postoperative demand for morphine, however, postoperative adverse reactions were more in this group. Although the incidence of postoperative pruritus in the ITM and TAPB groups was not significantly different, the incidence of PONV in the TAPB group was much lower than in the ITM group, which is also an advantage of the TAPB technology. A suitable approach to apply the advantages of TAPB to multimodal analgesia may provide an important direction to our future research.

This study has some limitations. First, the number of included studies were small and the number of samples was insufficient. With further relevant research, our understanding of ITM and TAPB will continue to expand. In addition, one study in our meta-analysis was from a developing area, and its outcome presents a high risk of bias. Second, pregnant women with a history of caesarean delivery were not excluded, and the multipara had used ITM or TAPB for postoperative analgesia after an earlier caesarean delivery. If the randomised treatment in this study is inconsistent with the pattern previously used in multipara, they may have doubts about the analgesic effect, thus breaking the blindness. Third, different doses and concentrations of local anaesthetics had been used in the studies, and corresponding drugs such as paracetamol and dexamethasone had also been used after surgery. Although these postoperative drugs had been used in both groups, the usage of these drugs increases the complexity of the present study. Finally, he detailed information about the technique used in TAPB was not available. TAPB is completed under either ultrasound or landmark positioning; however, different methods may produce different blocking effects, and exploration of these effects may be the focus of future studies.

## Conclusion

In conclusion, our meta-analysis indicated that ITM can produce superior analgesic effects in parturients after caesarean delivery and decrease the postoperative demand for morphine compared with TAPB. In addition, the incidence of itching was comparable between the groups, whereas the incidence of PONV was reported to be higher in the ITM group. Thus, TAPB could be recommended as a valuable analgesia option for patients who cannot use ITM for analgesia after caesarean delivery or those having a high risk of nausea and vomiting.

Further studies should focus on post-caesarean analgesia in developing countries. According to reports, most deliveries in the world occur in these countries [31]. However, due to the lack of personnel and economy in these countries, achieving optimum care and superior anaesthesia technology is difficult. More RCTs are needed to explore the most effective method for administering postpartum analgesia under unfavorable conditions such as lack of resources, which may also be the focus of our future study.

## Supplementary Information

**Additional file 1.** PRISMA checklist.pdf

## Data Availability

The datasets generated and analyzed during the current study are available from the corresponding author on reasonable request.

## Abbreviations

OR: Odds ratio
CI: Confidence interval
MD: Mean difference
PRISMA: Preferred Reporting Items for Systematic Reviews and Meta-Analyses
RCTs: Randomized controlled trials
MeSH: Medical Subject Heading
ITM: Intrathecal morphine
TAPB: Transversus abdominis plane block
PONV: Postoperative nausea and vomiting
